# First person – Michelle Stewart

**DOI:** 10.1242/dmm.039222

**Published:** 2019-02-22

## Abstract

First Person is a series of interviews with the first authors of a selection of papers published in Disease Models & Mechanisms, helping early-career researchers promote themselves alongside their papers. Michelle Stewart is first author on ‘Loss of *Frrs1l* disrupts synaptic AMPA receptor function, and results in neurodevelopmental, motor, cognitive and electrographical abnormalities’, published in DMM. Michelle is a scientific manager in the lab of Sara Wells at MRC Harwell Institute, Oxfordshire, UK, investigating neurobehavioural genetics, behaviour and ageing in mice.


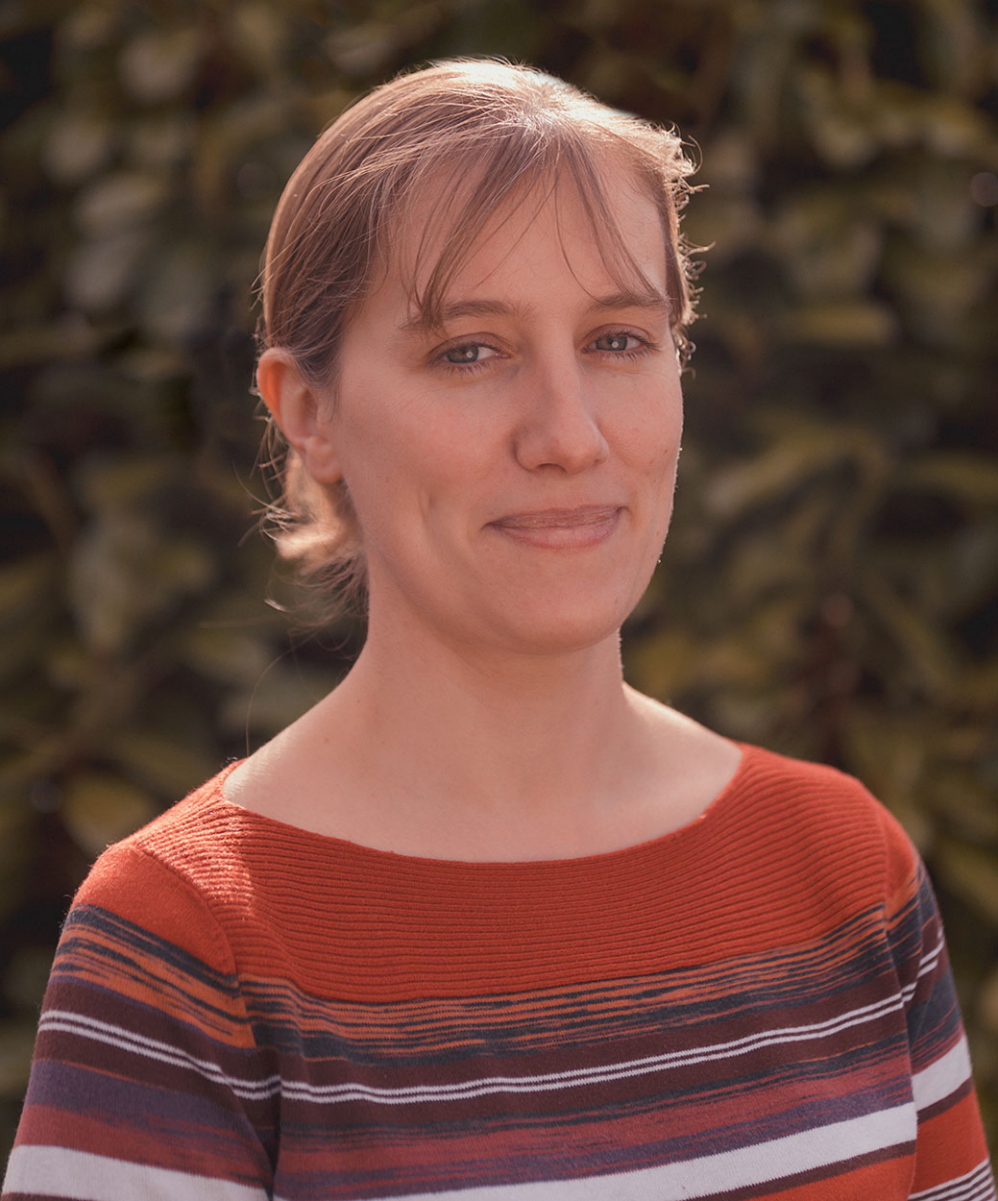


**Michelle Stewart**

**How would you explain the main findings of your paper to non-scientific family and friends?**

We found that when the gene ferric chelate reductase 1-like (*Frrs1l*) is turned off in mice, the mice show behavioural, motor and cognitive abnormalities, as well as abnormal electrical activity in the brain. Many of these phenotypes overlap with symptoms of human patients that have modifications to the same gene. Molecular analysis indicates that disrupting the function of *FRRS1L* reduces the overall quality and function of AMPA receptors in the brain. AMPA receptors are critical for conducting information between neurons. Without sufficient AMPA receptors, signalling in the brain can be severely disrupted and cause a variety of neurological phenotypes.

“Greater understanding of AMPA receptor function will provide increased insight into disease mechanisms and provide potential new drug targets.”

**What are the potential implications of these results for your field of research?**

The major components of AMPA receptors have been studied for over a decade. However, there is a growing number of previously uncharacterized proteins that are not part of the main core of the receptor but can have major impacts on the function, of which FRRS1L is one. Understanding the mechanism through which *FRRS1L* affects AMPA receptor function will give us greater insight into the fine control of these signalling mechanisms. AMPA receptor function is altered in many diseases, not only single gene mutations but also diseases of ageing such as Alzheimer's disease. Greater understanding of AMPA receptor function will provide increased insight into disease mechanisms and provide potential new drug targets.

**What are the main advantages and drawbacks of the model system you have used as it relates to the disease you are investigating?**

Mice have been an incredibly useful model for this study as they have allowed us to look at complex behaviour, motor function and electroencephalography in a mammalian system. There have been disadvantages, such as the high neonatal mortality of mice carrying homozygous deletions. The mechanism that causes this mortality is unknown, and the differences between mouse and human brain function at this early stage of life are not well understood. It is also difficult to model diseases that occur in humans throughout a longer lifespan, when mice only live a fraction of the time.

**What has surprised you the most while conducting your research?**

The thing that has surprised me most about this research is the resilience of the mammalian brain. Mice with homozygous deletions in *Frrs1l* that survive past the neonatal period have similar lifespans to normal wild-type mice. This is so surprising because the levels of AMPA receptors in the brain are so dramatically reduced. Our current knowledge of these signalling mechanisms implies that such a dramatic reduction would be incompatible with life; therefore, there must be significant parts of these mechanisms that we have yet to understand.

“There are many challenges in the area of behavioural genetics but one of the main issues is the behavioural tests themselves.”

**Figure:**
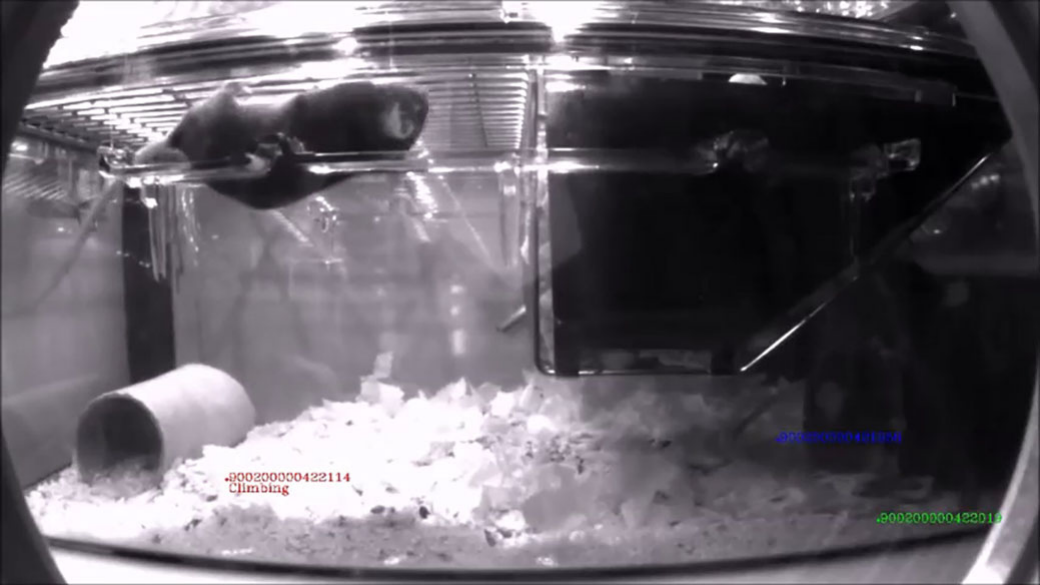
**Automated behaviour annotations capture phenotypes without experimenter intervention.**

**Describe what you think is the most significant challenge impacting your research at this time and how will this be addressed over the next 10 years?**

There are many challenges in the area of behavioural genetics but one of the main issues is the behavioural tests themselves. Many tests involve mazes or arenas in unfamiliar rooms, and they are often tested during the mouse sleep period and are likely to cause some level of stress. Human interaction with mice is known to impact results, as well as the time of day the test is done and the disturbance of moving mice to testing rooms. To address this, more and more tests are being developed in home-cage scenarios. In the home cage, we can look at more natural behaviour and reduce the variability inevitably introduced by human investigators. The hope is that this will also create more reproducible data and perhaps decrease animal numbers, as well as the severity of many procedures.

**What's next for you?**

Since finishing my PhD I have returned to my full-time role as scientific manager of the Mary Lyon Centre at the MRC Harwell Institute. Here, I am responsible for much of the day-to-day running of the animal unit, as well as advising on and coordinating many research projects for a variety of groups both based at MRC Harwell and across the UK.
